# Effect of Age, Education, and Bilingualism on Confrontation Naming in Older Illiterate and Low-Educated Populations

**DOI:** 10.1155/2014/970520

**Published:** 2014-02-12

**Authors:** Sameer Ashaie, Loraine Obler

**Affiliations:** Speech-Language-Hearing Sciences, CUNY Graduate Center, 365 Fifth Avenue, New York City, NY 10016, USA

## Abstract

We investigated the effects of age as well as the linked factors of education and bilingualism on confrontation naming in rural Kashmir by creating a culturally appropriate naming test with pictures of 60 objects. We recruited 48 cognitively normal participants whose ages ranged from 18 to 28 and from 60 to 85. Participants in our study were illiterate monolinguals (*N* = 18) and educated Kashmiri-Urdu bilinguals (*N* = 30). Hierarchical multiple regression revealed that younger adults performed better than older adults (*P* < 0.01) and the age effect was quadratic (age^2^). It also showed Age X Education and Age X L2 Speaking interactions predicted naming performance. The Age X Education interaction indicated that the advantages of greater education increased with advancing age. Since education is in the second language (L2) in our population, this finding is no doubt linked to the Age X L2 Speaking interaction. This suggests that L2 speaking proficiency contributed more to first language (L1) naming with advancing age.

## 1. Introduction

Performance on confrontation naming tests, in which individuals have to identify a visual stimulus representing an object or an action and then correctly label the stimulus aloud, has been linked to age, education, and bilingualism. According to most models of word production (e.g., [1, 2]), confrontation naming involves three stages: conceptual preparation, lemma retrieval (lexical selection of relevant names along with their semantic and syntactic properties), and lexeme retrieval (of phonological word-form information). Difficulties with confrontation naming can generally be attributed to problems with the lexeme retrieval stage and such difficulties are mostly age related in non-brain-damaged individuals [3, 4].

The majority of studies investigating confrontation naming in older adults have found that they perform significantly worse than younger adults (e.g., [5–9]) with decline in confrontation naming more pronounced after the age of 70 (see [10]). However several studies have not found age related differences in performance between older and younger adults (e.g., [11, 12]). The discrepancies could be attributed to varying research methods, age ranges, participant characteristics (e.g., education), and statistical methods, as pointed out by Goulet et al. [13]. Schmitter-Edgecombe et al. [14] actually found that older adults perform better on confrontation naming than younger adults. However, upon further examination of their data, they found that four items had generational familiarity that biased them towards older adults and could, thus, they argued, explain the finding. Though the majority of studies suggest age may have an effect on performance in confrontation naming tests, there are likely other variables that can account for performance differences.

An additional factor that has been found to influence naming is education [6, 7, 15–17]. Connor et al. [15] found that individuals with 16 years of education perform better on naming than those with 12 years of education. These studies and others (e.g., [4]) suggest that to a certain extent, higher education could increase performance on naming tests; however, the lower limits of educational levels for participants in these studies have been around the level of eight years of education.

Similar studies on naming in illiterates and low-educated individuals found that illiteracy and low educational levels decreased performance on confrontation naming tests [18, 19]. Manly et al. [19] found that literates with 0 to 3 years of education did significantly better than illiterates on a 15-item Boston Naming Test (BNT). However, Ganguli et al. [20], using three-dimensional models of objects instead of line drawings, did not report any difference in performance between literates and illiterates.

The results on confrontation naming tests with illiterate and low-educated participants can be attributed to a variety of factors including cultural relevance of test items and the participants' familiarity with task protocols as argued by Ardila [21] and Ganguli et al. [20]. Additionally, since line drawings (as compared to photographs or real objects) could be ambiguous or less recognizable for illiterates, the usage of such visual stimuli could make naming objects difficult [22]. To circumvent this problem, culturally appropriate tests have been used to assess naming performance in individuals across culturally diverse populations. Some of these studies, however, only included 8–15 items which makes it difficult to include items of varying lexical frequency. Studies with a small number of items may only include items with similar frequencies. This could potentially skew the results such that if only high frequency items are included, no difference between illiterates and literates is found. In contrast, if only low frequency culturally inappropriate items are used, illiterates will do worse than literates.

Education, like aging, cannot fully explain the variability found in performance of older adults on confrontation naming tests. One other factor, bilingualism, appears to play some role in an individual's performance on confrontation naming tests [23, 24].

Bilingualism has been found to adversely affect confrontation naming in younger and older adults in both L1 and L2 [23, 25, 26]. The bilingual disadvantage on naming tasks has been attributed to factors such as using each language less than a comparable monolingual, lower proficiency, smaller vocabularies in each individual language, and having a somewhat later age of acquisition of individual words.

A few studies have used a set of possible predictors of naming in L2 but not in L1. For example, Roberts et al. used L2 (English) percent usage, self-ratings of L2 auditory comprehension, and L2 verbal expression to investigate naming in L2. They did not address how proficiency in other L2 modalities (e.g., writing) influenced naming. It is, of course, possible that individuals in their study do not have different proficiency levels in different L2 modalities because they have more than 11 years of education and live in L2 speaking environments. Proficiency in speaking an L2 does not necessarily correlate with proficiency in reading and writing it.

We reasoned that individuals who have markedly lower education and live in L1-dominant environments have different L2 proficiency levels from participants like those of Roberts et al. [26]. For example, reading and writing proficiency could be confounded with higher education such that individuals who are highly educated are highly proficient in those modalities in either or both L1 and L2. Speaking, on the other hand, could be related with language usage and not necessarily with education. Indeed, even in their L1, Kashmiri, our participants were not literate; those who were literate among them were literate only in their school language L2, Urdu. Thus, in the current study we recorded individuals' L2 proficiency by individual modalities to determine the potentially different relations among proficiency levels in different L2 modalities and L1 naming scores.

In order to understand the effects of age, education, and bilingual modalities (reading, writing, and speaking) on confrontation naming in illiterates and low-educated individuals, we devised a culturally appropriate naming test to address the following questions: (1) Does performance on confrontation naming decline with advancing age in low-education populations? (2) What are the effects of education on confrontation naming in older illiterates and low educated individuals? (3) Does proficiency in different bilingual modalities impact confrontation naming differently?

## 2. Methods

### 2.1. Participants

Forty-eight participants were recruited for our study from a rural area (Mulphak) in Kashmir, India. The participants primarily had agricultural occupations. Half were male and half female with ages ranging between 18–28 and 60–85 years. The adult populations in rural areas of India typically have no memory or official record of their date of birth [27]. Out of 24 older participants (60–85) years, only 6 participants gave us an exact age. The remaining 18 participants gave us their ages in a 5-year range. To further confirm the validity of their age and age ranges, older participants and their family members were asked to estimate the participant's age at the time of major historical events (e.g., India's partition) and their family history (e.g., marriage). The practice of confirming older participants' ages to personal and historical events is consistent with the methodology used in other studies (e.g., [27]). For the analyses reported below, we used as each participant's age either the precise age, when they gave us one, or the upper limit of the 5-year range. A second set of analyses using the lower limit of the range for those 18 participants gave us the same findings so we do not report them here.

Eighteen participants were illiterate, with no years of formal schooling. An illiterate was defined as “someone who cannot, with understanding, both read and write a short, simple statement on his or her everyday life” [28]. The ability to write was assessed by asking the participants to write 2-3 sentences in Urdu about their typical day. The ability to read was assessed by asking the participants to read aloud a paragraph in Urdu from the local newspaper. Those who could do so, even with some errors, were deemed literate. Thirty participants were literate, with education ranging from 1 to 10 years (see Table 1). The educated participants were Kashmiri-Urdu bilinguals because the medium of education in rural areas of Kashmir is Urdu. The educated participants speak in their native language, Kashmiri, but cannot read nor write Kashmiri. They can, however, speak, read, and write Urdu.

### 2.2. Materials

#### 2.2.1. Kashmiri Naming Test (KNT)

Items for the Kashmiri Naming Test were selected based on information from individuals living in the rural area where the confrontation naming test was conducted. Prior to running the confrontation naming study, since no frequency measure of Kashmiri words exists, we spent time with two families in the area where testing would take place (approximately 10 members ranging in ages 16–90) and enquired about picturable items that we deemed to be of low, middle, and high frequency. Sixty items that all the family members of different age levels and education ranges agreed upon as belonging to low (*N* = 20), middle (*N* = 19), and high (*N* = 21) word frequencies were used in the KNT (see Appendix A). Furthermore, the 60 nouns chosen for the KNT would have been acquired in daily life outside the school. In the test itself, the items were not ordered according to difficulty. Since this was the first time illiterates were in a testing situation, we did not want fatigue to influence naming of low frequency items. Furthermore, actual color photographs of the items were used in the KNT because illiterates have difficulty recognizing black and white line drawings [29].

#### 2.2.2. Proficiency Questionnaire

A proficiency questionnaire (Appendix B) was designed to investigate educated participants' L2 proficiency and usage. L2 proficiency was investigated by asking the educated participants to rate their Urdu speaking, reading, and writing proficiencies on a 0–4 scale (0 = no ability, 1 = poor, 2 = functional, 3 = good, and 4 = excellent). We also asked which language was their regularly spoken language (for all it was Kashmiri) and whether they were educated in Kashmiri or Urdu in case some participants might have been educated in Kashmiri although that is not standard in this region; none were.

#### 2.2.3. Adapted Mini-Mental State Exam (MMSE; [30])

The Mini-Mental State Examination (MMSE; [30]) is a widely used neuropsychological test to screen for cognitively impaired or demented individuals. MMSE is a brief and reliable test used in a large number of epidemiological studies [31] and consists of 12 questions which measure various cognitive domains such as memory, orientation, and praxis. This test can be translated and adapted into various languages to make it culturally valid for the populations being tested [32, 33].

There is no official translation of MMSE into Kashmiri and individual doctors may translate the English MMSE into Kashmiri for use with patients. However, relying on an English MMSE for our population would be problematic since it includes items that are culturally inappropriate. For example, the English MMSE asks participants to name hospitals and buildings but rural areas in Kashmir do not have major buildings or hospitals. Thus, we took as a base for our Kashmiri version of the MMSE a Hindi version of the MMSE since it was designed for a similar rural population (see [32] for details). The initial translation was done by an educated Kashmiri speaker and then checked by a professional translator for translation accuracy. The final version of the Kashmiri MMSE was also checked by a local neurologist. Our aim for using the Kashmiri MMSE was to screen participants for any cognitive impairment since we were only interested in naming in healthy older adults. The cutoff score we selected in advance for all participants was 19 out of 30 as Ganguli et al. [32] had used in their study of Hindi speakers. All participants tested met this criterion.

### 2.3. Procedure

The testing was conducted by a native speaker of Kashmiri in the participants' homes. Before the testing session began, the participants were informed about the tests they would be asked to complete. Since this was the first time the illiterates were in a testing situation, extra care was taken to make them feel comfortable with the investigator and his research assistant. For example, it was the first time some of the older individuals had seen the laptop and they had to be made comfortable looking at the computer screen (e.g., by comparing the computer to a television).

After the informed-consent procedure was carried out, the participants were administered the literacy test and then the adapted Kashmiri MMSE. After a 10-minute break, the KNT was administered to the participants. The participants were shown pictures of the 60 items on the KNT one by one on a laptop computer. The participants' responses to the items were recorded on a scoring sheet. If the participants did not give an answer in 30 seconds, a phonemic cue was provided. A phonemic cue was also provided if the participant described the item but could not name it. The overall correct responses only reflected items that were named correctly without the use of phonemic cues. After the KNT was administered, the educated participants were asked to fill out the L2 proficiency and usage questionnaire.

## 3. Results

The internal consistency of the 60 items that comprise KNT was measured by obtaining Cronbach's alpha coefficient. The analysis revealed a high alpha coefficient (Cronbach's *α* = 0.895) indicating that items on the KNT are internally consistent.

Descriptive statistics and Pearson correlations are reported in Table 2. In all the analyses below, we only analyzed data from 47 participants because one of the participants was omitted as an outlier on the naming test. Age and KNT scores were significantly correlated, *r* = −0.57, *P* < 0.01. There was also a significant correlation between education and L2 speaking *r* = 0.75, *P* < 0.01, education and L2 writing *r* = 0.68, *P* < 0.01, and education and L2 reading *r* = 73, *P* < 0.01.

Hierarchical multiple regression (HMR) was used to test how age, education, L2 speaking, L2 reading, and L2 writing predicted KNT scores (Table 3). In order to avoid problems of multicollinearity we centered age, education, L2 speaking, and L2 reading and writing at their respective means (see Table 2). Since most studies have found that age and education significantly predict naming scores, we entered them in step 1 of our analysis. The results of step 1 indicated that these two independent variables (age and education) accounted for 35% of the variance, which was statistically greater than zero, *F*(2,44) = 12.05, *P* < 0.01. Age was the only statistically significant independent variable (*β* = −0.57, *P* < 0.01). In step 2, the 3 bilingualism self-rated proficiency modalities (L2 speaking, L2 reading, and L2 writing) were entered into the regression equation. The change in variance accounted for (Δ*R* ^2^) was 1% and not statistically significant, *F*(3,41) = 0.20, *P* > 0.05. Thus, the bilingual proficiency modalities (L2 speaking, L2 reading, and L2 writing) did not themselves significantly predict naming scores in L1.

**Figure 1:**
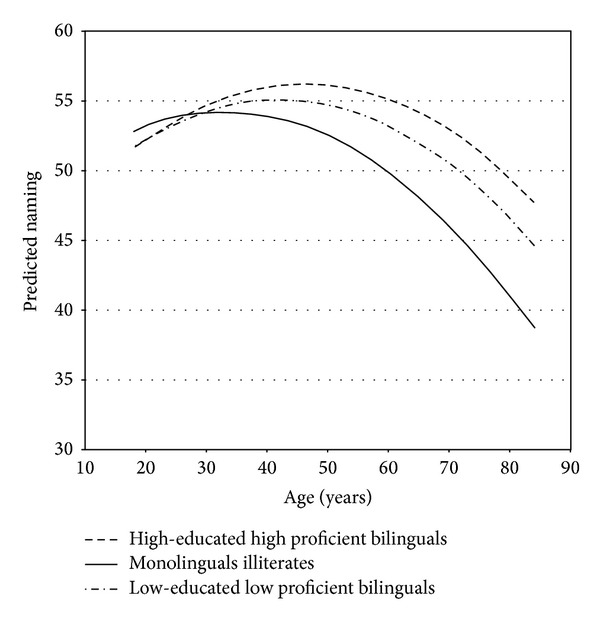
Estimated nonlinear effect of age (age^2^) for high education high proficient bilinguals (education = 10, L2 proficiency variables = 3), low-educated low proficient bilinguals (education = 4, L2 proficiency variables = 2), and monolingual illiterates.

**Figure 2:**
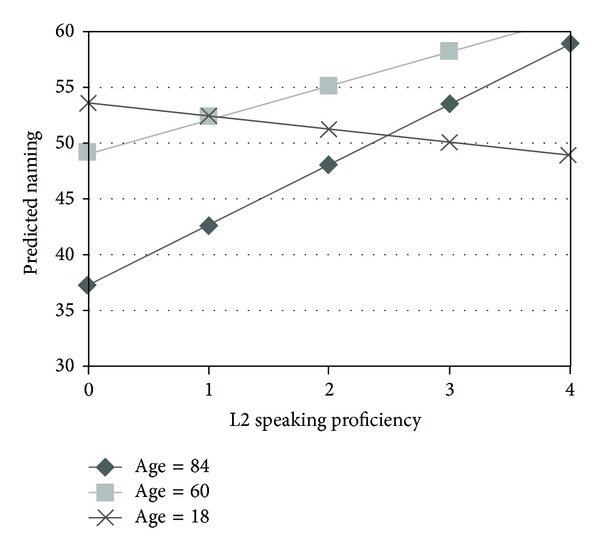
Estimated effect of L2 speaking proficiency on naming (mean values for education, L2 reading, and writing proficiencies).

We also investigated whether the age effect was quadratic, entering age^2^ into step 3 of our analysis (Table 3). The quadratic effect of age was significant (*β* = −0.30, *P* < 0.05) as illustrated in Figure 1. Age X Education interactions were entered into step 4 of our analysis (Table 3). The Age X Education interaction was a significant (*β* = 0.27, *P* < 0.05) predictor of naming such that the advantages of greater education increased with advancing age. Age X L2 Speaking, Age X L2 Reading, and Age X L2 Writing interactions were then entered in step 5 of our regression analysis (Table 2). Only the Age X Speaking L2 interaction significantly predicted (*β* = −0.55, *P* < 0.05) naming such that the proficiency of L2 speaking contributed more to L1 naming with advancing age (Figure 2).

As well, we investigated whether lexical frequency affects KNT scores and if they are modulated by age, education, L2 speaking, L2 reading and L2 writing measures. A repeated measures ANOVA indicated that naming scores differed across different lexical frequencies, *F*(2,52) = 34.5, *P* < 0.05. Pairwise comparisons revealed that naming scores were the lowest for the low frequency words. There was no significant interaction between lexical frequencies and age, education, or any of the L2 variables.

**Figure 3:**
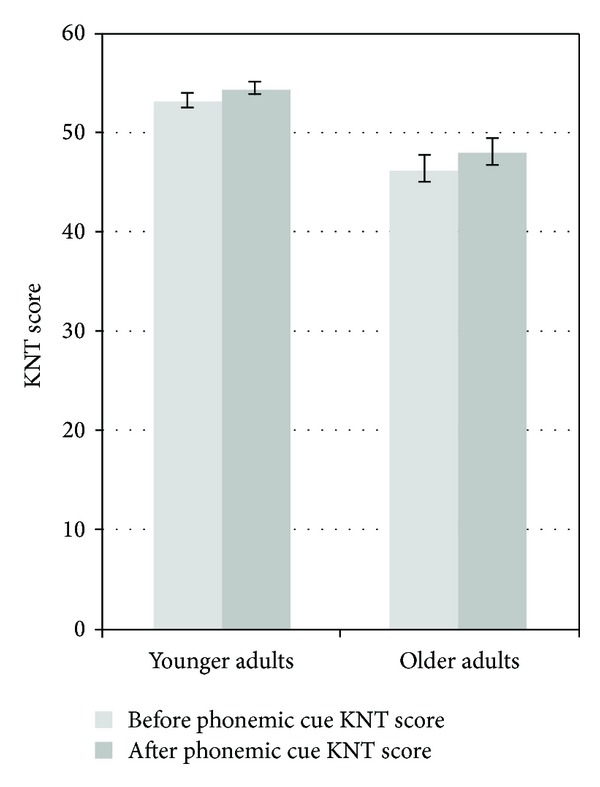
Mean KNT scores before and after phonemic cues.

Additionally, we investigated whether participants' scores significantly improved after the use of phonemic cues. A mixed model ANOVA indicated that KNT scores improved after the use of phonemic cues *F*(1,45) = 26.47, *P* < 0.05 for both the older and younger adults (see Figure 3). There was no interaction between age and KNT scores *F*(1,45) = 0.57, *P* > 0.05 such that the phonemic cues did not differentially benefit one group more than the other.

## 4. Discussion

The BNT and its various versions are commonly used in cross-national studies to test confrontation naming in older adults (e.g., [8]). The BNT was developed for a North American population and cross-national studies using the BNT have found that some of the items are not culturally appropriate for other populations [34]. For example, items such as “unicorn” on the BNT are not culturally relevant to rural areas of Kashmir and thus it was not appropriate to include them. Thus, we devised a culturally appropriate 60-item naming test to address whether age, education, and bilingual modalities (reading, writing, and speaking proficiencies) affect confrontation naming.

Our study indicates that naming declines with advancing age in our population. The decline is curvilinear (Figure 1) in that confrontation naming scores increase in early adult years and then decrease with an accelerating decline, particularly after the age of 70 (as in [35]). Of course, since our study does not have confrontation naming scores for participants aged 29–59, the pattern our model estimates for that age range should be interpreted with caution. Nevertheless we note that our model provides striking parallels to the findings of the Language in the Aging Brain Laboratory for a higher-educated monolingual older-adult population (e.g., [15, 36]). In addition, the inflection point in our illiterate participants is similar to that of Goral et al. [4] for their higher-educated (16 years) and less-educated (12 years) participants alike, around age 36.

The age-related decline observed in our study can be explained by the Transmission Deficit Hypothesis [37]. Specifically, this model involves networks of processing units divided into lexical, phonological, and semantic nodes which are connected to each other. During the process of word retrieval, the concept of the word in the semantic system activates a lexical node which in turn primes the phonological node so that the correct word is produced. The amount of priming is dependent upon strength of connection between the nodes. If the connection between the nodes is weak, lexical retrieval of the words is difficult [4]. According to the TDH model, older adults face lexical retrieval difficulties due to a decreased amount of priming resulting from a weakening of connections between different nodes. Our results show that after the age of 70, naming decline is rapid, suggesting that the connection between lexical, semantic, and phonological nodes is rapidly weakening, consistent with the TDH model.

The TDH model has also been used to explain the Tip-of-the-Tongue (TOT) phenomenon which occurs in older adults due to weak connections between lexical and phonological nodes [3]. The TOT phenomenon occurs when individuals are certain that they know the name of the target or can either define or else provide partial phonology of the target but cannot retrieve the full name unless some sort of cue is provided [3]. Indeed, a mixed model ANOVA indicated that the phonemic cues improved scores of younger and older participants equally (*P* > 0.05, see Figure 3).

In contrast to aging, education per se did not have an effect on L1 naming in this study. This is consistent with a number of studies [9, 20, 38–40]. Furthermore, our results are not surprising, because we only included items on our confrontation naming test that were part of the culture and individuals of all educational backgrounds had acquired them in early life. According to Juhasz [36] and others (e.g., [41]) words learned earlier in life are processed with greater accuracy and faster than those learned later. Moreover, as a result of the way items were selected for the KNT, it is possible that the lexical target nouns were not the ones that were learned in schooling. Perhaps the education effect found in some but not all studies of naming and aging occurs only when school-learned items (e.g., protractor, compass) are among the targets.

However, we found an Age X Education interaction such that older illiterates performed worse than older educated adults, even though we had selected target items that are the ones learned not in school but in daily life. Our results were consistent with Welch et al. [16]. The interaction between education and age-related decline on naming in older adults could also be explained by a model developed by Capitani et al. [42]. They proposed three different possible outcomes for interaction between age-related decline and education on neuropsychological tests: (1) parallelism: the age-related decline runs the same course in different educational groups (i.e., no interaction); (2) protection: the age-related decline is attenuated in well-educated participants; (3) confluence: the initial advantage of well-educated groups in middle age is reduced in later life. Our results support the protection hypothesis, even though our better-educated older adults do not have the same degree of high education as those in the Capitani et al. [42] study.

With regards to whether bilingual proficiency affects naming, we did not find an individual bilingual modality effect. It is possible that individual bilingual modalities did not affect confrontation naming because our population was atypical of the bilingual populations generally studied in the bilingualism literature. Our bilingual participants did not read and write in their L1 but only spoke it. Also, the lexical items of their rural home environment may not overlap with the academic L2 taught at school, so perhaps there was no interference from being bilingual on the items tested in the KNT. Since the participants were dominant in their spoken L1, it is possible that there was no distraction from their L2 which could inhibit L1 production as Kroll et al. [43] posited that it should. However, we did find an Age X L2 Speaking interaction such that older bilinguals did better than older monolingual adults on L1 confrontation naming.

Though bilingualism has been found to attenuate other age-related cognitive decline (e.g., [44]), it has been reported to adversely affect picture naming in both younger and older adults (e.g., [25, 26]). Studies that found bilingualism impacted picture naming adversely (e.g., [26]) did not specifically investigate whether bilingualism interacted with age as we did here. Furthermore, in these studies, the effect of L2 speaking proficiency specifically on naming was not investigated. Our results suggest that speaking a second language may attenuate age-related decline in picture naming in the first language. We did not find an L2 reading or writing interaction with age, which suggests that it is L2 speaking ability that matters the most. Bialystok et al. [44] found a bilingual advantage for working memory tasks in older bilingual adults compared to monolinguals. Our data suggest that it would be important to look at specific bilingual modalities to determine how proficiency in them affects different tasks. It is possible that speaking ability was a major determinant in Bialystok et al. [44] study. Nevertheless, our results further extend the bilingual advantage to age-related naming decline and refine our understanding to focus on L2 speaking proficiency.

Both the Age X Education interaction and Age X L2 Speaking interaction that we found could be explained by the cognitive reserve hypothesis. The cognitive reserve hypothesis states that individual differences in how tasks are processed reflect differences in how the brain copes with brain injuries and age-related changes [45]. According to this hypothesis, educated individuals and bilinguals may have greater neural reserve (brain or cognitive networks) as a result of engagement of cognitive processes in acquiring new information and in using more than one language. The increased neural reserve in these individuals serves to protect them against the negative effects of aging and potential brain injuries [46, 47]. It is possible that being educated and being proficient in a second language increased the older individuals' neural reserve and this benefited them in their old age.

Our study also addresses the suggestion of Gollan et al. [48] that the benefits of bilingualism may only be conferred on those with low education. Gollan et al. [48] found that higher degrees of bilingualism delay the onset of Alzheimer's disease only in individuals with less than 12 years of education. The authors suggest that higher-educated individuals did not benefit from bilingualism because at higher educational levels the power of cognitive reserve has reached its upper limit and thus there is no further benefit of bilingualism on cognitive reserve. Our study is consistent with this claim in that none of our participants had greater than 10 years of education and the combination of bilingualism and education appears to have benefited them in picture naming in their first language because they had not reached the posited upper limit of cognitive reserve. However, it is important to keep in mind that further studies are needed to better understand the nature and limits of cognitive reserve.

As others have observed (e.g., [21]), it is challenging to test illiterate populations because the participants may not understand the concept of testing and may never have been in a testing environment. We dealt with this challenge by testing individuals in their homes and providing substantial training on the task before testing began. Moreover, we created a culture-appropriate set of photographs of items whose names ranged from high to low frequencies to permit success on the task yet provide the opportunity for variable performance. Our test also included only those items whose names would have been acquired outside of school, thus eliminating the possibility of having educationally biased items.

A conceptual challenge we faced was the confounding of education and bilingualism in our study, although we were fortunate that even individuals in the same education brackets ranged in their L2 proficiency. Further indication of the confounding of education and bilingualism in our study is seen in the result from HMR reported in Table 2. Note that when the Age X L2 proficiency interaction was added at step 5, the Age X Education term was no longer significant, strongly suggesting that L2 proficiency and education, not surprisingly in this population, measure similar things. Only a study in a population where L2 proficiency and years of education can be dissociated could resolve the question of the relative contributions of each to naming in aging and, one would hope, to cognitive reserve more generally.

Our efforts at confronting the challenges permit us to report on naming in younger illiterates whereas prior studies have only found illiterates among older adults (e.g., [9]), showing that illiteracy along with monolingualism does not result in poorer L1 naming accuracy in 18–29 year olds. These results support the claim that naming declines most precipitously after age 70 and permit us to argue that young illiterates and monolinguals are not at a disadvantage compared to their age-matched educated and bilingual counterparts when it comes to lexical retrieval. However, it is possible that younger illiterates are slower in naming than their younger educated and bilingual peers. Further investigation is needed to understand whether education benefits naming latencies at any age.

In light of the challenges of testing a low-educated rural population among whom age must be estimated based on memory for salient historical events, it is all the more impressive that our results revealed age-related naming declines very similar to those reported among higher-educated Western populations. Furthermore, our study was also unique in that even our educated participants had low ranges of education (1–5 and 6–10 years) and thus we were able to determine that even in a population with few years of education by Western standards, more education and L2 proficiency offset naming decline in older age.

We note that even though KNT was designed for a healthy population, it could also be employed or adapted (e.g., by reducing the number of items or by excluding the low frequency items) to investigate the naming performances of individuals with aphasia and individuals suspected of having dementia. Furthermore, since the KNT has items of varying frequency, one could also investigate how errors in different types of aphasia and dementia might be related to word frequency levels.

## Figures and Tables

**Table 1:** Participant characteristics.

		*N* = 10		*N* = 7		*N* = 7	
		Illiterates (0 education)		Low educated (1–5 years)		High educated (6–10 years)	
		*M *	SD	*M *	SD	*M *	SD
	MMSE	25.40	3.86	22.85	3.57	27.42	1.81
Age 18–28	KNT Score	53.90	3.54	52.5	4.27	52.85	3.62
	L2 speaking proficiency	0	0	1.71	1.38	2.85	1.06
	L2 reading proficiency	0	0	.71	.95	1.85	1.34
	L2 writing proficiency	0	0	.71	.95	2.28	1.38

		*N* = 8		*N* = 8		*N* = 7	
		*M *	SD	*M *	SD	*M *	SD

	MMSE	24.37	3.15	26.50	1.60	26.14	1.77
Age 60–85	KNT Score	42.25	4.92	47.12	8.57	50.14	3.80
	L2 speaking proficiency	0	0	.87	1.12	1.85	1.06
	L2 reading proficiency	0	0	1.25	1.75	2.42	1.27
	L2 writing proficiency	0	0	1.25	1.75	2.57	1.39

*Note*. One participant was omitted as an outlier (age = 85, MMSE = 28, L2 speaking proficiency = 4, L2 reading proficiency = 4, L2 writing proficiency = 4, and KNT score = 19).

**Table 2:** Descriptive statistics for variables in the hierarchical multiple regression.

Variable	*M *	SD	*Q*_1_	Mdn	*Q*_3_	100 × Pearson correlation					
						1	2	3	4	5	6
(1) Age (years)	47.40	25.62	24	28	75	—					
(2) Education	4.15	3.71	0	5	8	−3	—				
(3) L2 speaking	1.11	1.36	0	1	2	−18	75**	—			
(4) L2 writing	.96	1.37	0	0	2	12	68**	68**	—		
(5) L2 reading	1.04	1.46	0	0	2	8	73**	71**	96**	—	
(6) naming	49.85	6.32	47	51	56	−57**	18	20	4	9	—

*Note*. *N* = 47 participants (1 omitted as an outlier). *Q*_1_ = 1st quartile, and *Q*_3_ = 3rd quartile.

**P < .01.

**Table 3:** Results from the five-step hierarchical multiple regression, predicting naming.

Predictor	Step 1		Step 2		Step 3		Step 4		Step 5	
	*b *	**β**	*b *	**β**	*b *	**β**	*b *	**β**	*b *	**β**
Intercept	49.85**		49.85**		54.16**		52.85**		54.73**	
Age^a^	−.14	−.57**	−.15	−.58**	−.12	−.48**	−.12	−.46**	−.09	.37**
Education^a^	.28	.16	.30	.18	.09	−.04	.09	.05	.07	.04
L2 speaking^a^			−.46	−.11	−.05	−.01	.55	.12	1.85	−.39
L2 writing^a^			−1.12	−.24	−1.11	−.24	−1.84	−.40	−5.40	−1.16
L2 reading^a^			1.38	.32	1.53	.35	1.73	.40	4.40	1.00
Age squared^b^					−.01	−.30*	−.01	−.21	−.01	−.28*
Age × education^b^							.02	.27*	.00	.02
Age × L2 speaking^b^									.10	.55*
Age × L2 writing^b^									−.20	−1.12
Age × L2 reading^b^									.16	.94

*R* ^ 2^	.35		.36		.44		.49		.57	
*F* for *R* ^2^	12.05**		4.70**		5.22**		5.43**		4.70**	
Δ*R* ^2^			.01		.07		.05		.07	
*F* for Δ*R* ^2^			.20		5.44*		4.20*		2.06	

*Note*. *N* = 47 participants (1 omitted as an outlier). *b* = estimate of unstandardized partial regression coefficient. **β** = estimate of standardized partial regression coefficient.

^a^Centered at sample mean (see Table 1). ^b^Square or product computed from centered variable(s).

*P < .05. **P < .01.

**Table 4:** Kashmiri Naming Test.

Item name	
(1) “Bael”	Shovel
(2) “Khrav”	A type of wooden slipper
(3) “Nadur”	Lotus stem
(4) “Bugin”	A type of earthen piggybank
(5) “Rabab”	A guitar-like musical instrument
(6) “Traam”	A special plate on which four people can eat
(7) “Kukur”	Chicken
(8) “Pheran”	Woolen cloak worn in winter
(9) “Wukhul”	A small stone pestle
(10) “Hangul”	Kashmiri stag
(11) “Phot”	A large wooden basket
(12) “Tsong”	Earthen lamp
(13) “Kangir”	Earthen fire-pot to keep warm in winter
(14) “Balteen”	Bucket
(15) “Tsery”	Dried apricots
(16) “Radio”	Radio
(17) “Aal”	Gourd
(18) “Booni”	Chinar tree
(19) “Grayti”	A large stone grinder used in farms
(20) “Bushkaab”	A type of plate in which men and boys eat
(21) “Daan”	A specific type of clay hearth with an oven
(22) “Latsul”	Broom
(23) “Muhul”	A large pestle used in farms
(24) “Doyn”	Wooden churner
(25) “Takar”	Basket
(26) “Martoor”	Claw hammer
(27) “Birbatayn”	Wooden toy
(28) “Haak”	Kale
(29) “Tsuchwur”	A type of bagel
(30) “Dandabrush”	Toothbrush
(31) “Dukaeer”	Scissors
(32) “Naaw”	Boat
(33) “Haaput”	Bear
(34) “Yander”	Spinning wheel
(35) “Karakuli”	A type of hat worn by men
(36) “Kang”	A clay pot for used for burning coal
(37) “Tsestan”	Needle
(38) “Pambach”	Lotus seed head
(39) “Tash”	A utensil used for draining water when washing hands
(40) “Chumta”	Tongs
(41) “Aalbayn”	A specific type of plow
(42) “Zoon”	Yolk
(43) “Pulhor”	Grass slipper
(44) “Hayr”	Ladder
(45) “Haydar”	Mushroom
(46) “Tumbaknar”	A drum-like musical instrument
(47) “Khat”	Sheep
(48) “Chilim”	Clay pipe for preparing tobacco
(49) “Kaynz”	A type of plate from which women and girls eat
(50) “Nalka”	Tap
(51) “Satut”	Hoopoe
(52) “Palas”	Plier
(53) “Droot”	Grass sickle
(54) “Dul”	A type of earthen pot for liquids
(55) “Kangin”	A type of wooden comb
(56) “Manzul”	Wooden crib
(57) “Watne-gur”	Wooden baby walker
(58) “Nai”	Flute
(59) “Gantebayr”	Kite
(60) “Anyut”	A type of earthen lid

## Appendices

### A.

See Table 4.

### B. Proficiency Questionnaire

Please select your level of education:

Grade 1:   □
Grade 2:  □
Grade 3:  □
Grade 4:  □
Grade 5:  □
Grade 6:  □
Grade 7:  □
Grade 8:  □
Grade 9:  □
Grade 10:  □

Please select your language of education:

Urdu:   □
Kashmiri:  □

Spoken language:

Urdu:   □
Kashmiri:  □

What was the age of acquisition of your languages?

Kashmiri___________ Urdu___________
What language do you speak the most? ___________
Where do you speak Urdu? ___________
Do you read Urdu? ___________
How much Urdu do you read? ___________
Do you write in Urdu? ___________
How much Urdu do you write? ___________
When is the last time you spoke in Urdu? ___________
When is the last time you read in Urdu? ___________
When is the last time you wrote in Urdu? ___________
Please rate your spoken Urdu proficiency (0 = no ability, 1 = poor, 2 = functional, 3 = good, and 4 = excellent).
___________
Please rate your written Urdu proficiency (0 = no ability, 1 = poor, 2 = functional, 3 = good, and 4 = excellent).
___________
Please rate your reading proficiency in Urdu (0 = no ability, 1 = poor, 2 = functional, 3 = good, and 4 = excellent).
___________
